# Measuring the efficacy of standard and novel disinfection methods on frequently used physical therapy equipment: a 2-phase prospective randomized controlled trial

**DOI:** 10.1017/ice.2024.101

**Published:** 2024-10

**Authors:** Bobby G. Warren, Aaron Barrett, Amanda Graves, Guerbine Fils-Aime, Jennifer Edelschick, Jolinda Cullinan, Diandrea McCottter, Nicholas A. Turner, Deverick J. Anderson

**Affiliations:** 1 Duke Center for Antimicrobial Stewardship and Infection Prevention, Durham, NC, USA; 2 Disinfection, Resistance and Transmission Epidemiology (DiRTE) Lab, Duke University School of Medicine, Durham, NC, USA; 3 Division of Infectious Diseases, Duke University Medical Center, Durham, NC, USA; 4 Physical and Occupational Therapy, Duke University Medical Center, Durham, NC, USA

## Abstract

**Background:**

Frequently used physical therapy (PT) equipment is difficult to disinfect due to equipment material and shape. The efficacy of standard disinfection of PT equipment is poorly understood.

**Methods:**

We completed a 2-phase prospective microbiological analysis of fomites used in PT at our hospital from September 2022 to October 2023. For both phases, study fomites were obtained after usage and split into symmetrical halves for sampling. In phase 1, sides were sampled following standard disinfection. In phase 2, sides were randomized 1:1 to intervention or control. Samples were obtained before and after the intervention, a disinfection cabinet using Ultraviolet C (UV-C) and 6% nebulized hydrogen peroxide. We defined antimicrobial-resistant clinically important pathogens (AMR CIP) as methicillin-resistant staphylococcus aureus (MRSA), Vancomycin Resistant Enterococcus (VRE), and Multidrug resistant (MDR)-Gram-negatives and non-AMR CIP as methicillin-sensitive staphylococcus aureus (MSSA), Vancomycin sensitive Enterococcus (VSE), and Gram-negatives. Three assessments were made: 1) contamination following standard disinfection (phase 1), 2) contamination postintervention compared to no disinfection (phase 2) and, 3) contamination following standard disinfection compared to postintervention (phase 1 vs phase 2 intervention).

**Results:**

The median total colony-forming units (CFU) from 122 study fomite samples was 1,348 (IQR 398–2,365). At the sample level, 52(43%) and 15(12%) of samples harbored any clinically important pathogens (CIPs) or AMR CIPs, respectively. The median CFU was 0 (IQR 0–55) in the intervention group and 977 (409–2,547) in the control group (*P* < .00001).

**Conclusion:**

Following standard disinfection, PT equipment remained heavily contaminated including AMR and non-AMR CIPs. Following the intervention, PT equipment was less contaminated and harbored no AMR CIPs compared to control sides supporting the efficacy of the intervention on difficult-to-disinfect PT fomites.

## Introduction

Around 721,000 healthcare-associated infections (HAIs) occur annually in the United States, leading to approximately 75,000 patient deaths within hospitals.^1–3^ Many of these HAIs are attributed to multidrug-resistant organisms, which impact around 2 million individuals in the U.S. each year and result in adverse patient outcomes and fatalities.^2^

Pathogens responsible for HAIs are frequently discovered on various healthcare surfaces and equipment.^4–6^ Pathogens present on these surfaces and the healthcare environment also contribute to transmission among patients and healthcare personnel.^7–10^ Consequently, disinfection of medical equipment plays a crucial role in preventing HAIs and the transmission of pathogens.

While numerous studies pinpoint medical equipment as pathogen reservoirs, there has been a limited focus on investigating physical therapy (PT) equipment. The examination of physical therapy equipment is important due to the wide array of equipment types employed, ranging from standardized to non-standardized, and the challenges posed in disinfecting them, particularly those with intricate shapes or materials, such as physical therapy balls with deep ridges.

We performed a randomized controlled trial and microbiological analysis to describe the contamination of physical therapy equipment after standard disinfection and to determine the efficacy of a novel disinfection device on difficult-to-disinfect items.

## Methods

### Objectives

Our primary objective was to determine the effectiveness of an enhanced disinfection strategy, a disinfection cabinet using Ultraviolet C (UV-C) and 6% nebulized hydrogen peroxide by volume, on frequently used and difficult-to-dinfect PT equipment. Our secondary objective was to describe and characterize the contamination of frequently used PT equipment after undergoing standard disinfection.

### Patient consent statement

This study was reviewed by the Duke University Health System Institutional Review Board and received an “exempt” status.

### Study setting

We completed a microbiological analysis of and randomized controlled trial involving equipment used in adult or pediatric PT at Duke University Health System, Durham, NC.

### Study protocol

Our study involved two phases. First, we evaluated baseline contamination present on used physical therapy equipment following standard disinfection. Second, we performed a randomized controlled trial to compare residual contamination of equipment for a novel disinfection strategy compared to standard disinfection. Standard disinfection was defined as the use of a disinfectant wipe on fomites following PT patient treatment: Oxivir Tb wipes (Diversey, Fort Mill, NC, USA) for patients on contact precautions and the Sani-Cloth for all others (PDI, Woodcliff Lake, NJ, USA). For both phases, physical therapy equipment, hereafter “study fomites,” was obtained from PT staff returning from patient treatment in an inpatient unit and split into two symmetric halves, left and right, for sampling. Clinical data was collected from the corresponding patient for fomites that were used on a single patient since the fomites last disinfection. Clinical data was not collected on some fomites as they were used unit-wide since the fomites last disinfection, such as walkers, since they were not linked to a specific patient.

**Figure 1.**
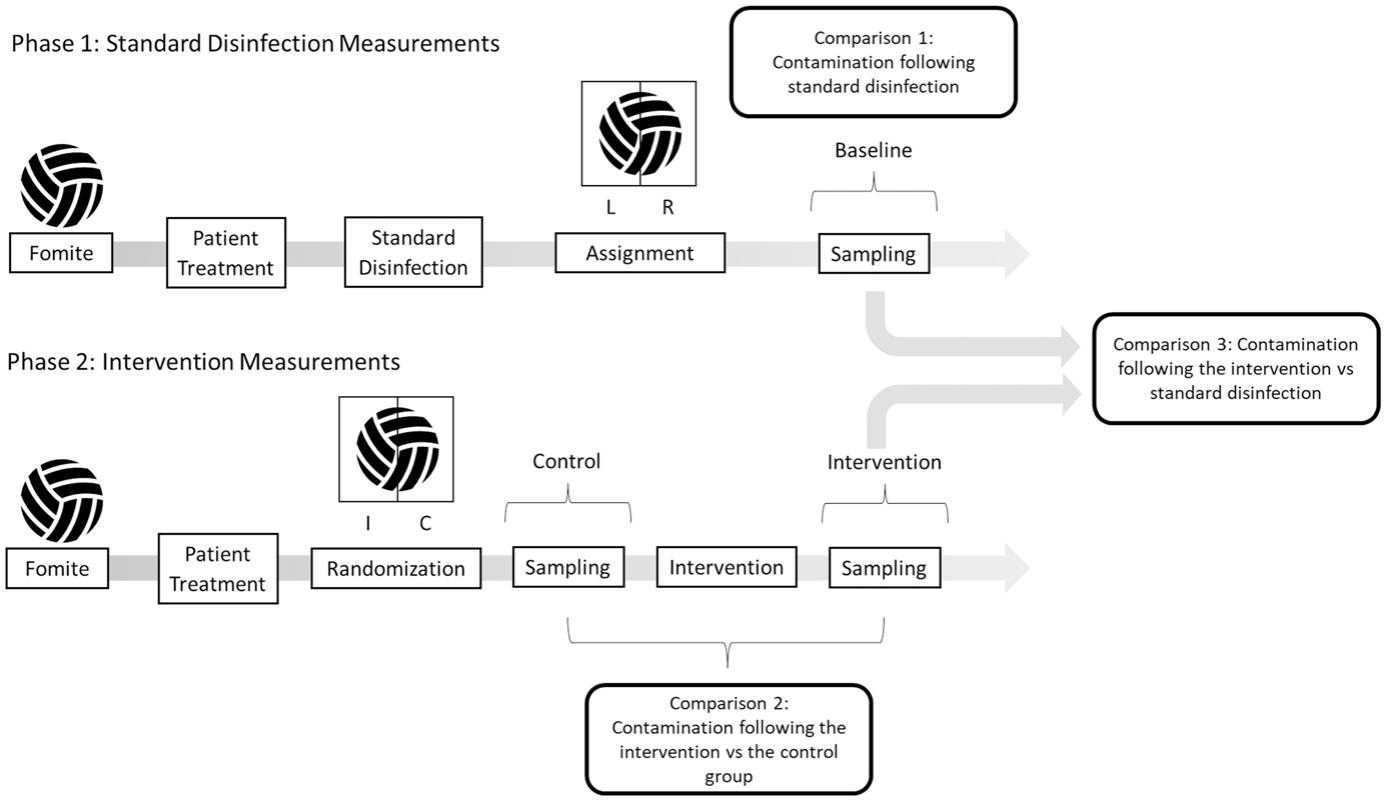
Study workflow and comparisons.

During the trial, study fomite sides were randomized 1:1 to intervention and control. Standard disinfection was not completed before sampling. For each piece of equipment, the control fomite side was sampled before intervention exposure. The fomite then underwent the intervention and was disinfected using the standard disinfection cycle of the PURitALL 3030L disinfection cabinet. The PURitALL 3030L utilizes UV-C in combination with 6% nebulized hydrogen peroxide by volume inside a stainless-steel casing for disinfection over the course of an estimated 15-to-20-minute runtime. The intervention fomite side was then sampled after the intervention exposure. The above approach allowed us to evaluate three sets of data: prior to any disinfection (Phase 2 control), following standard disinfection (Phase 1 “baseline”), and following the intervention (Phase 2 intervention) (Figure 1).

### Microbiological methods

Samples were taken with premoistened cellulose sponges and processed using the stomacher technique.^11^ Sponges were placed in stomacher bags with 45 mL phosphate-buffered saline with 1% Tween 20 and homogenized for 60 seconds at 260 revolutions per minute (RPM). Homogenates were then centrifuged at 3200 RPM for 15 minutes, and all but approximately 5 mL of the resulting supernatant was discarded. Then, each sample’s volume was measured to control for variability and rehomogenized via vortex. A total of 200 μL of the final homogenate was plated onto study media and incubated at 37°C for 24 hours.^5^ Homogenates were plated on Trypticase soy agar with 5% sheep’s blood for general bioburden, Bile-esculin agar for *Enterococcus spp.*, Mannitol salt for *S. aureus*, and MacConkey agar for Gram-negative species. Gram-negative isolates were speciated with 16S rRNA gene sequencing and were assessed for carbapenemase gene presence via polyemerase chain reaction (PCR) with primers designed for Klebsiella Pneumoniae Carbapenemase (KPC), New Delhi metallo-beta-lactamase 1 (NDM-1), OXA-48, IMP, and VIM.^12–14^

We defined clinically important pathogens (CIPs) as *S. aureus* (methicillin-resistant S. aureus [MRSA] or methicillin-sensitive *S. aureus*), *Enterococci spp.* (vancomycin-resistant *Enterococcus* [VRE] or vancomycin-sensitive *Enterococcus*), *Acinetobacter spp*., *Pseudomonas spp*., and Enterobacterales of interest such as *Escherichia coli* and *Klebsiella spp*. We defined antimicrobial-resistant clinically important pathogens (AMR CIP) as MRSA, VRE, and Multidrug resistant (MDR)-Gram-negatives and non-AMR CIP as methicillin-sensitive staphylococcus aureus (MSSA), Vancomycin sensitive Enterococcus (VSE), and Gram-negative species. Study fomites were grouped as 1) walking aids (walkers or canes), 2) pediatric toys, 3) balls (medicine, dodge, etc.), and 4) other (Foam roller, sliding board, etc.).

### Outcomes

The primary outcome is the amount of bioburden, measured in colony-forming units (CFUs), on intervention sides of fomites in phase 2. Our secondary outcomes were the bioburden on fomites following standard disinfection and the proportion of samples positive for CIPs. Outcomes were compared between left and right fomite sides for standard disinfection measurements and control and intervention sides for intervention measurements.

### Data analysis

The Wilcoxon ranked sum test was used to compare CFU measurements and the Z score proportionality test was used to compare proportions of samples with CIPs. *P* < .05 was considered significant, all statistical tests were 2-tailed, and all testing was completed using R software (R Foundation for Statistical Computing, Vienna, Austria).

## Results

From September 2022 to October 2023, 257 fomites and a total of 514 samples were analyzed. Clinical data was collected on patients who underwent treatment with study fomites. However, due to unit-wide use of fomites in adult wards, clinical data were primarily only available for pediatric patients. Thus, data was available for 116 patients from 122 (47%) fomites. Data from fomites with no clinical data was not excluded from analysis. Overall, the median age was 2 (IQR: 1–11), 66 (57%) were female, 20 (17%) had active infections, and 24 (21%) were on contact precautions.

### Phase 1 - Standard disinfection measurements

A total of 61 fomites from 47 patients were analyzed following disinfection by PT staff. Of the study patients, 24 (51%) were female, 13 (27%) had active infections, and 15 (32%) were on contact precautions. Overall, no differences were observed between sides. The median total colony-forming-units (CFU) from the 122 study fomite samples was 1348 (IQR 398–2365): 468 (IQR 161–1230) for the left side study arm and 540 (IQR 102–1221) for the right (*P* = .45).

At the fomite level, 27 (44%), 5 (8%), 15 (25%), and 7 (11%) of 61 fomites harbored any CIPs, only AMR CIPs, only non-AMR CIPs, or both AMR and non-AMR CIPs, respectively. At the sample level, 52 (43%), 15 (12%) and 37 (30%) of 122 samples harbored any CIPs, AMR CIPs, or non-AMR CIPs, respectively. Notably, CIPs between fomite sides were similar. Generally, balls were the most contaminated study fomites (2237 CFU [IQR 1425–2658]); walking aids were most frequently contaminated with any CIPs (26 [72%]), AMR CIPs (8 [22%]) and non-AMR CIPs (15 [47%]) (Table 1).

**Table 1.** Bioburden in colony-forming units (CFU) after use of physical therapy equipment after standard disinfection

	Overall N = 122 n (IQR)	Left N = 61 n (IQR)	Right N = 61 n (IQR)	*P*
Sample CFUs, median (IQR)	1,348 (398–2,365)	468 (161–1,230)	540 (102–1,221)	0.45
Toys, N = 42	586 (172–725)	228 (112–460)	96 (48–350)	0.19
Walking aids, N = 36	1,076 (374–2,320)	660 (198–1,260)	638 (251–1,231)	0.16
Therapy Balls, N = 32	2,237 (1,425–2,658)	813 (613–1,233)	918 (732–1,628)	0.44
Other, N = 12	909 (428–1,619)	350 (309–715)	325 (119–1,138)	0.94
Total clinically important pathogens, No./total No. (%), N = 122				
Total	52 (43)	23 (38)	29 (48)	0.27
AMR CIPs	15 (12)	7 (11)	8 (13)	0.78
Non-AMR CIPs	37 (30)	16 (26)	21 (34)	0.33
Toys, N = 42				
Total	5 (12)	2 (9)	3 (14)	0.65
AMR CIPs	1 (2)	0	1 (5)	
Non-AMR CIPs	4 (9)	2 (9)	2 (9)	
Walking aids, N = 36				
Total	26 (72)	14 (78)	12 (67)	0.62
AMR CIPs	8 (22)	4 (22)	4 (22)	
Non-AMR CIPs	18 (50)	10 (56)	8 (44)	
Therapy Balls, N = 32				
Total	21 (66)	7 (44)	14 (88)	0.06
AMR CIPs	6 (19)	3 (19)	3 (19)	
Non-AMR CIPs	15 (47)	4 (25)	11 (69)	
Other, N = 12				
Total	0	0	0	1
AMR CIPs	0	0	0	1
Non-AMR CIPs	0	0	0	1

### Phase 2 - Intervention measurements

A total of 196 fomites from 69 patients were analyzed. The median age of study patients was 2 (IQR: 1–10), 42 (61%) were female, 7 (10%) had active infections, and 9 (13%) were on contact precautions. The median CFU from the 392 study fomite samples was 0 (IQR 0–55) in the intervention group and 977 (409–2,547) in the control group (*P* < .00001).

At the sample level, 3 (2%) and 0 (0%) of 196 samples in the intervention group and, 37 (19%) and 7 (4%) of 196 samples in the control group harbored any CIPs or AMR CIPs, respectively (*P* = < .00001, .008, respectively) (Table 2). For the control group, generally, “other” were the most contaminated study fomites (2,052 [IQR 579–5,398]), and walking aids were most frequently contaminated with any CIPs (23 [23%]), AMR CIPs (5 [5%]) and non-AMR CIPs (30 [30%]) (Table 2). Lastly, the disinfection cabinet’s average running time was 18 minutes.

**Table 2.** Bioburden in colony-forming units (CFU) after use of physical therapy equipment after enhanced disinfection and after standard disinfection

	Control N = 196	Enhanced Disinfection (Intervention) N = 196	Standard Disinfection N = 122	*P, Int vs Ctl* (Primary analysis)*, Int vs SD*
Sample CFUs, median (IQR)	977 (409–2,547)	0 (0–55)	527 (117–1,218)	<0.00001, <0.00001
Walking Aids, N = 98, 98, 36	1,088 (600–2,430)	0 (0–55)	782 (245–1,498)	<0.00001, <0.00001
Toys, N = 50, 50, 42	452 (265–1,1110)	0 (0–13)	152 (56–462)	<0.00001, <0.00001
Balls, N = 26, 26, 32	1,755 (412–15,452)	0 (0–150)	838 (673–1,498)	<0.00001, <0.00001
Other, N = 22, 22, 12	2,052 (579–5,398)	0 (0–293)	350 (119–1,146)	0.0001, 0.007
Total clinically important pathogens, No./total No. (%), N = 196, 196, 122				
Total	37 (19)	3 (2)	52 (43)	<0.00001, <0.00001
AMR CIPs	7 (4)	0 (0)	15 (12)	0.008, <0.0001
Non-AMR CIPs	30 (15)	3 (2)	37 (30)	<0.00001, <0.00001
Walking Aids, N = 98, 98, 36				
Total	23 (23)	1 (1)	5 (12)	<0.00001, 0.001
AMR CIPs	5 (5)	0 (0)	1 (2)	0.02, 0.1
Non-AMR CIPs	18 (18)	1 (1)	4 (9)	<0.00001, 0.006
Toys, N = 50, 50, 42				
Total	6 (12)	0 (0)	26 (72)	0.01, <0.0001
AMR CIPs	0 (0)	0 (0)	8 (22)	1, 0.001
Non-AMR CIPs	6 (12)	0 (0)	18 (50)	0.01, <0.0001
Balls, N = 26, 26, 32				
Total	4 (15)	1 (1)	21 (66)	0.2, <0.0001
AMR CIPs	0 (0)	0 (0)	6 (19)	1, 0.02
Non-AMR CIPs	4 (15)	1 (1)	15 (47)	0.2, <0.0001
Other, N = 22, 22, 12				
Total	4 (18)	1 (1)	0 (0)	0.2, 0.5
AMR CIPs	2 (9)	0 (0)	0 (0)	0.2, 1
Non-AMR CIPs	4 (18)	1 (1)	0 (0)	0.2, 0.5

### Intervention measurements compared to standard disinfection measurements

The overall median bioburden was significantly lower in intervention measurements (0 CFU [IQR: 0–55]) compared to standard disinfection (527 [117–1,218] (*P* = < .0001). The proportion of samples harboring CIPs was significantly lower in intervention measurements (3 [2%] of 196 samples) compared to standard disinfection (52 [43%] out of 122 samples), (*P* = < .0001). These differences remained when stratifying the data by fomite or AMR groupings (Table 2).

## Discussion

Healthcare equipment is frequently contaminated with CIPs and can play a role in in-hospital transmissions.^15–21^ However, some types of shared medical equipment can be difficult to disinfect due to material and shape. Therefore, novel strategies beyond wipes and other mechanical methods are needed. Our objectives were to characterize the residual contamination on shared physical therapy equipment after standard disinfection and the efficacy of a novel device that utilizes nebulized 6% hydrogen peroxide by volume to overcome equipment material and shape difficulties. Following standard disinfection, used PT equipment remained heavily contaminated, confirming the belief that PT equipment is difficult to disinfect via standard disinfection likely due to fomite shape and material exacerbating known difficulties with wipe-based disinfectants such as required contact times (Oxivir TB: 2 minutes, Sani-Cloth: 1 minute)Additionally, left-, and right-side fomite divisions had similar bioburdens during baseline testing, suggesting that this sampling model may be helpful for resolving case-mix issues in future studies evaluating disinfection strategies. Following the intervention, used PT equipment was markedly less contaminated and harbored no AMR CIPs compared to control sides. Lastly, when comparing the intervention to standard disinfection, sides receiving the intervention had significantly lower bioburden and CIP presence than standard disinfection.

Limited research on the contamination of physical therapy equipment has been published. To date, most research in this area has been in response to an outbreak or investigating a specific fomite of concern. For example, Aljadi et al. investigated interferential and electrical stimulation equipment and observed that 68.3% of samples contained any bioburden^22^. Similarly, Spratt Jr et al described the contamination of therapeutic ultrasound equipment and found that 52.7% of ultrasound gel bottle tips contained any bioburden, and 3.6% were positive for MRSA.^23^ Similar to our study, Gontjes et al. completed a prospective study of environmental contamination in six nursing homes including each facility’s rehabilitation gym. They found that 7.7% of rehabilitation gym equipment samples harbored at least one AMR CIP, a finding similar to our finding of 12%. The increased prevalence in our study may be due to our use of sponges for sample collection opposed to Gontjes et al’s used of swabs, which increases the sample area tested or the study setting and population.^24^

Our study has limitations. First, the adherence to disinfection of fomites in our Phase 1 assessment of baseline contamination following standard disinfection was not measured so our comparisons are really to the current use of standard disinfection, not its pure efficacy. However, these devices were retrieved from the area where equipment is placed after it has been processed and disinfected as part of routine procedures. While this approach could have led to increased bioburden in the standard disinfection measurements, it is also representative of “real world” clinical processes and care. Second, only 112 (44%) of 257 fomites had associated clinical data collected. Therefore, our ability to discuss the impact of different patient characteristics on subsequent contamination of equipment is limited. Third, our results are only generalizable to settings where wipes are currently used to disinfect items of odd shape and material. Fourth, the intervention was tested with an absence of physical cleaning prior to disinfection. Although the results still indicate the intervention’s efficacy, all fomites were wiped, and therefore cleaned, during standard disinfection so fomites in phase 2 were likely not long removed from a recent cleaning. Therefore, our results are only applicable to non-soiled fomites, and future work is needed on the impact of cleaning prior to disinfection with the intervention. However, the amount of pediatric and adult patients was roughly equal in all three comparisons for fomites with clinical data so the impact should be minimal. Fourth, the impact of reduction of contamination on fomites, in general, and on our study’s fomites, on patient outcomes is unclear. Finally, both pediatric and adult patients were grouped in analyses and could have differences in bioburden impacts on study fomites.

In summary, persistent contamination with important pathogens was observed on physical therapy equipment, even after standard disinfection practices were performed. This finding may be due to the material and shape of items and the resulting difficulty of applying disinfectant. However, the use of the disinfection cabinet led to significantly less bioburden, fewer CIPs, and the elimination of AMR CIPs compared to the control arm and standard disinfection. These findings support the efficacy of the disinfection cabinet on difficult-to-disinfect fomites and warrant future studies examining the device’s efficacy on difficult-to-disinfect items in areas where transmission and outbreaks are more likely.

## Supporting information

Warren et al. supplementary material 1 Warren et al. supplementary material
