# Supplementary material for: Measuring the efficacy of standard and novel disinfection methods on frequently used physical therapy equipment: a 2-phase prospective randomized controlled trial
Source: Infect Control Hosp Epidemiol. 2024 Jul 26;45(10):1219–24. doi: 10.1017/ice.2024.101 (PMC11611508; doi:10.1017/ice.2024.101)
Supplement: Warren et al. supplementary material 1 — Warren et al. supplementary material [file S0899823X24001016sup001.docx]

Supplementary file 1. Descriptions of study fomites and sample procedures

**Walking Aids**

Included fomites and material: Walkers and canes constructed of aluminum and rubber.

Splitting: Walkers and canes were split into left and right sides from the orientation of someone using the walker straight down the middle.

Sampling procedures: The entire surface of each side was carefully sampled except for the rubber bottoms and wheels that made contact with the floor.

**Toys**

Included fomites and material: A plastic piggy bank in the shape of a pig, with a door on its right side and plastic pop-up toy.

Splitting: Pigs were split into left and right sides from the orientation of someone looking at the door on the pig’s side to include the inside section of the toy. Pop-up toys were split from the orientation of someone using the toy.

Sampling procedures: The entire surface of each side was carefully sampled including the inside of the pig toy to target difficult to disinfect areas and fomite.

**Balls**

Included fomites and material: Medicine balls (cloth textiles and rubber), dodge balls (rubber), spiky (rubber) and grip balls (rubber), etc.

Splitting: Balls were split by locating the inflation port, marking a line above and below the port to maintain orientation, and assigning left and right according to the orientation of the port and line.

Sampling procedure: The entire surface of each side was carefully sampled avoiding overlap between fomites sides.

**Other**

Included fomites and material: Sliding board (wooden with polyurethane coating), infant’s feeding chair (rubber), and foam rollers (foam)

Splitting: Sliding boards and the infant’s chair were split into left and right sides from the orientation of someone sitting on them. Foam rollers were split into left and right from the orientation from someone using it – laid on a surface where it could roll and you put your hands on each circle shaped side, the left-hand half was assigned left and vice versa.

Sampling procedure: The entire surface of each side was carefully sampled avoiding overlap between fomites sides. Portions of the infant’s feeding chair that made contact with the ground were not sampled.
